# Duplicating Vulcanite Plates

**Published:** 1879-08

**Authors:** W. R. Holmes


### ARTICLE V.

### *Duplicating Vulcanite Plates.*

BY W. R. HOLMES.

In casting about for a subject, I have not sought a very scientific one, as the above heading plainly shows, but chosen one which may be of practical benefit to some practitioner.

My mode of duplicating a plate, on vulcanite base, may not be entirely original, but I have no doubt to some it will be new, and when learned will be of value to them. Each of us can learn something, one from the other. Each dentist stumbles, as it were, in the practice upon some particular mode of performing a piece of work, which may in itself appear a trifle, yet is invaluable for that special purpose.

Every dentist also has his own peculiar way of operating upon the natural organs of mastication, his own peculiar way of supplying these organs by artificial means when lost, some particular way of taking an impression, some peculiar and original way of obtaining the articulation of artificial dentures, some particular way of vulcanizing, some peculiar way of finishing; each and all of us have our hobbies; we acquire them we know not how, nor why. All these are little things, it is true, but to us very essential to insure ease and facility in operating and to secure success.

Let us, then, exchange ideas, whether they be as "grains of sand or seeds of pearl." Life is made up of little things.

It is the atom that makes the great world. It is the chemical elements that make cells, cells that make tissues, tissues that make "the human form divine."

The teeth are small things and yet how vastly important for health, beauty and comfort! It is apparently a small thing to operate upon them, and yet how much skill is required to preserve them successfully. It is the little virtues of men and women that make them the great men and women of the world, and the kindness we exhibit toward each other that helps to make this life pleasant and agreeable. It is so in science, it is so in mechanism, it is so in dentistry. It is a little thing to say, "keep your excavators and pluggers sharp, and see that they are in order before you commence upon a tooth," but upon that depends the perfection and finish of your filling. It is a little thing to say prepare well the corners and edges of your cavity, but upon that depends the durability and permanence of your plug. It is a little thing to say "be sure that your fillings are kept entirely free from moisture," but upon that depends the thorough welding or uniting of the particles of gold. It is a little thing to say be patient, be thorough, be careful, be strong, and give every one who employs you your best gifts and services, but upon that depends your success as a dentist. It is a little thing to say be clean, be modest, be manly, but upon that depends your acceptability as a dentist. These things are to the dentist what the rain and dew are to the plants and flowers; they serve to make them flourish and grow, and become ornaments to society and the world. So it is in artificial dentures; the little things are the final adjustments or articulation—to see that every tooth impinges against every other tooth so that mastication is perfect—to see that the shade of the upper artificial teeth are the exact shade of the under natural teeth—to see that in the full sets the size, contour and shade are perfectly adapted to the complexion and suitable to the general appearance of the patient. They, too, are little things, and yet how necessary to obtain that naturalness so much to be desired by the patient and the ambitious dentist.

After the dentist, by the observance of all these minor points, as well as the larger ones, has made a beautiful set of artificial dentures, and they are inserted in the mouth, if his work has been well done and the patient seems pleased, he gazes upon his handiwork with feelings of pride and exultation, as some master artist when, upon canvas, he has painted the gorgeous mimicry of nature; or some sculptor, when he has hewn from the rough marble a perfect model of nature. It is apparently a little thing to exhibit so much pride about, and yet the dentist should be pardoned for such an indulgence. If he has pride and ambition he feels that he has accomplished a great good, and supplied a loss that would be unbearable did not he, by his art, come forward to the rescue. The work finished, the patient goes home delighted and happy in his possession, and the dentist rejoices at his success.

Time—that rude unobserver of rights and privileges—passes, and some day that beautiful piece of workmanship is carelessly allowed to fall and be broken to pieces; or perchance, in the mouth, from some cause, is made to snap asunder, or a block of teeth broken. The plate is returned to the dentist, and he looks and finds his beautiful work a mere wreck of its former perfectness. What must now be done? Usually, a new plate is made, or the parts are placed together with wax, the plate invested in plaster in the flask, separated, holes drilled through, new rubber put in, and the parts brought together, vulcanized and finished. Then again, the rubber solder is used to make the rubber stick to the old plate. This makes a good patch job, but the former beauty and strength is gone. How can this be remedied? I have fallen upon the following plan or *modus operandi:* When a plate is presented, it matters not how badly broken, so the pieces are all on hand, we unite the broken parts together with wax and model nicely, then mixing some plaster and pouring it into the lower section of the flask, press the plate into it, teeth looking downward, allowing the plaster to come up above the gums of the teeth; we slope it off towards the rim of the flask, then varnish and oil are applied to the plaster, the inner side of plate being kept perfectly clean and free from plaster, varnish or oil. The second section of flask being now placed in position, the plaster is mixed and poured in and the flask carefully shaken until we are assured that every part of the plate is thoroughly covered. It is then allowed to harden perfectly. The vulcanizer is now filled half full of water and heat applied ; when steam is raised, the flask is put therein. It is allowed to remain until it is thoroughly heated by a considerable amount of steam. The flask is then quickly removed, and rapidly, though carefully separated, and as soon as this is done, the rubber, which is now found in a semi-soft state, must be pulled hurriedly, though with some degree of care, out and away from the teeth and pins. It usually yields from them as readily as gutta percha base plates, and leaves the teeth intact, and pins clean and nice for packing. If it does not yield readily, heat it again and remove as before described. After this is accomplished, cut ways for excess of rubber; pack, vulcanize and finish in usual way. I have had such fine success by this method that I now never attempt to mend a plate in the ordinary way, but always duplicate. I find it requires less time, is decidedly more satisfactory, and usually for fee charge, half price of a new plate. It is every whit as perfect as a new one. If a section of teeth is broken off I fit a new one in place, model with wax, imbed in plaster, and proceed as before described.

There are no doubt some to whom this method will be new, and if I have succeeded in giving them any new ideas on the subject of duplicating vulcanite plates, I will feel that I have been fully compensated for the efforts of this paper.—*Dental Luminary.*

